# Citrus threat huanglongbing (HLB) - Could the rootstock provide the cure?

**DOI:** 10.3389/fpls.2024.1330846

**Published:** 2024-02-09

**Authors:** Rafaqat A. Gill, Xianglian Li, Shuo Duan, Qian Xing, Ralf Müller-Xing

**Affiliations:** ^1^ Lushan Botanical Garden, Chinese Academy of Sciences, Jiujiang, China; ^2^ College of Life Science, Nanchang University, Nanchang, China; ^3^ China-USA Citrus Huanglongbing Joint Laboratory (A Joint Laboratory of The University of Florida’s Institute of Food and Agricultural Sciences and Gannan Normal University), National Navel Orange Engineering Research Center, Gannan Normal University, Ganzhou, Jiangxi, China

**Keywords:** Citrus fruit trees, *Candidatus Liberibacter* (*CL*), Citrus greening, grafting, root-to-shoot transport, tRNA-like structures (TLSs), CRISPR/Cas9, heritable transgene-free genome editing

## 1 Introduction

Citrus production faces numerous environmental challenges including the devastating huanglongbing (HLB) disease. HLB, also known as citrus greening, affects health, growth and fruit quality of Citrus plants (Wang, 2019). Citrus crops have a long history of cultivation as grafted trees on selected rootstock cultivars that can improve tree performance and provide some degree of resistance to HLB (Shokrollah et al., 2011; Bowman and Albrecht, 2020; Bowman et al., 2021). Recently, several transgenic approaches have made significant progress in combating HLB. However, public acceptance of genetically modified (GM) crops is very low, and many consumers prefer to eat GM-free food (Lucht, 2015). In this article, we explore the potential of different approaches to combat HLB by grafting non-transgenic scions on transgenic and non-transgenic rootstocks.

## 2 HLB, a pathogen-triggered immune disease

HLB is caused by the infection with the phloem-colonizing bacterium *Candidatus Liberibacter* (*CL*) resulting in phloem cell death that is the key for most HLB symptoms. Early symptoms of the HLB disease include a yellow blotchy pattern in leaves and yellow shoots. In later stages, infected trees bear small-sized, deformed, and poorly colored (greening) fruits. A recent study demonstrates that HLB is an immune-mediated plant disease triggered by *CL* infection that stimulates systemic and chronic immune response in phloem tissues, including reactive oxygen species (ROS) production, callose deposition and induction of immune-related genes (Ma W. et al., 2022). HLB pathogens are transmitted by Asian citrus psyllid (*Diaphorina citri*), an insects of the Psyllidae family (Nadarasah and Stavrinides, 2011). The life cycle of the Psyllids depends on the Citrus plants, whose young flushes are the food and nursery of the Psyllid nymphs, while the adult insects suck sap from the phloem. As primary endosymbionts of the Psyllidae, *CL* bacteria are contained within the bacteriome, an interspecies organ comprising a close association of insect and bacterial cells (Ramsey et al., 2015). The Psyllids transmit the *CL* bacteria by sucking the phloem sap of Citrus plants. The propagation of the *CL* bacteria depends entirely on the Psyllid and the Citrus hosts. All attempts to culture *CL* failed until recently, making research and combat of HLB very challenging (Ha et al., 2019).

## 3 Limited effects of non-transgenic Citrus rootstock on HLB symptoms in scions

While some Citrus rootstocks, such as *C. trifoliata* and some of its hybrids, are tolerant to *CL* infection as seedlings, this tolerance to infection generally does not result in a large improvement in the HLB tolerance of trees, when the rootstock is grafted with a *CL*-sensitive scion, such as sweet orange (Bowman and Joubert, 2020). Therefore, using HLB-tolerant rootstocks only partially increases the overall tree tolerance to *CL* in the field. However, this approach can significantly enhance yield, fruit quality, and tree size (Bowman et al., 2016). Rootstocks can also improve the quality of fruit juice in HLB-affected Citrus by enhancing metabolites such as flavonoids and terpenoids (Huang et al., 2020). Ground application of manganese to rootstocks enhances root density and deteriorates *CL* populations, promoting healthier trees and improves fruit yield (Zambon et al., 2019). Hence, the choice of HLB-tolerant rootstocks and enhanced nutrition can significantly contribute to a better overall tree tolerance. Still, achieving full resistance to HLB requires additional strategies.

## 4 Recent advances in combating HLB by transgenic approaches involving salicylic acid-dependent defense pathways

**Figure 1:**
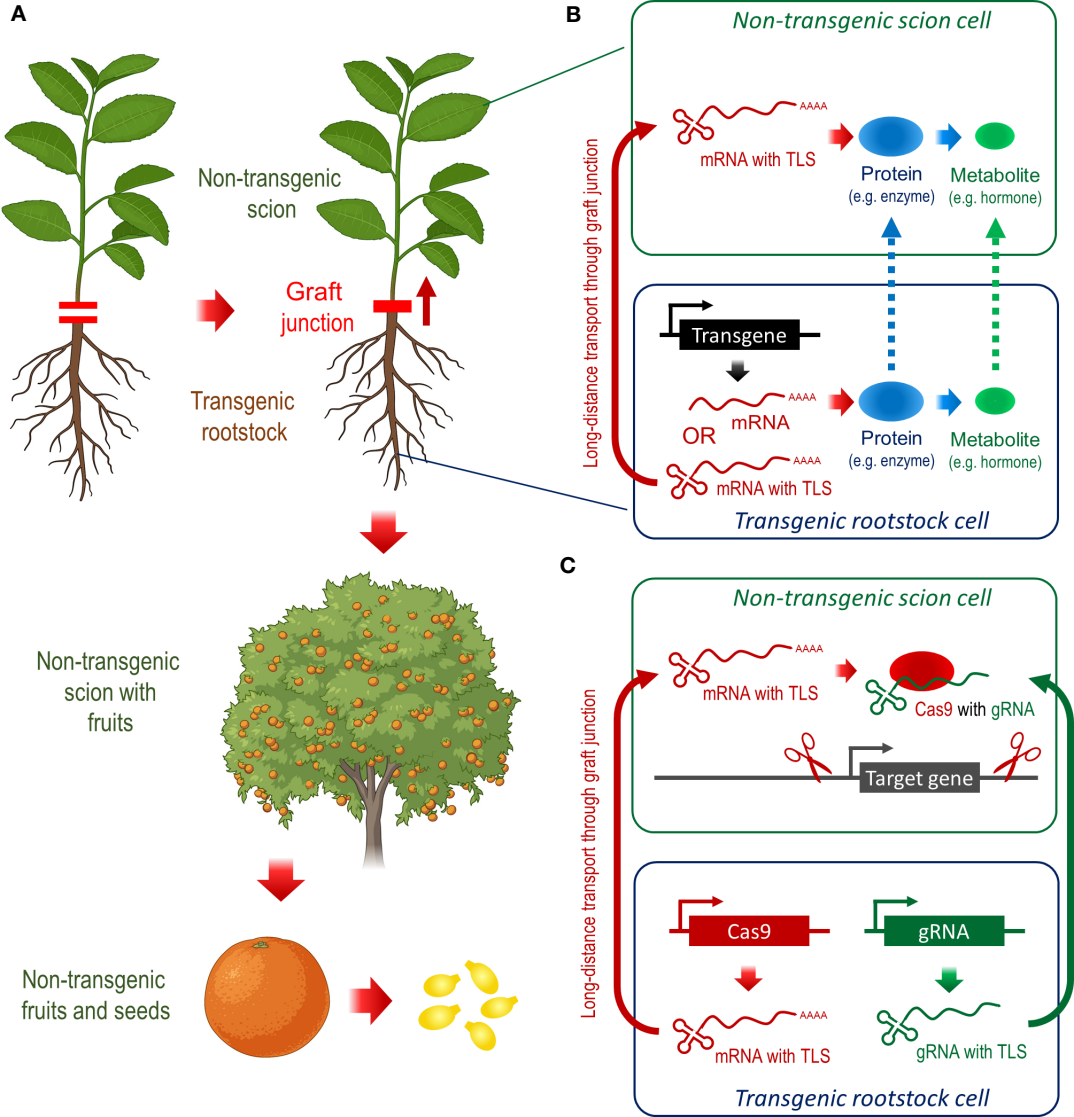
Transgenic rootstocks can provide desirable traits in non-transgenic scions of fruit trees, which is a powerful tool to combat HLB in Citrus. **(A)** Grafting of non-transgenic scion to transgenic rootstocks can improve traits of scion, fruits, and other products, which remain transgene-free. **(B)** Theoretically, mRNAs, proteins, and/or secondary metabolites can transmit the transgene function from the rootstock to the scion, but the fusion of RNAs to TLS sequences seems to be the most promising approach for this task. **(C)** Heritable transgene-free genome editing by grafting and TLS-dependent mobility of components of the CRISPR/Cas9 genome editing system.

Root-originated RNAs, proteins, and secondary metabolites can be transported via the vascular system to the shoot. This opens the doors for approaches using transgenic rootstocks to combat HLB disease in non-transgenic scions (**Figure 1A**). Recent studies have demonstrated that the exogenous application of the phytohormone salicylic acid (SA) to Citrus rootstocks and overexpression of components of the SA signal transduction pathway can improve the resistance to HLB and other Citrus pathogens. In general, SA signaling is the core of the plant defense mechanisms against pathogens, which can activate locally hypersensitive response (HR) and systemic acquired resistance (SAR) (Gao et al., 2015). Phloem-specific and constitutive expression of *Arabidopsis NONEXPRESSOR OF PR1* (*NPR1*), which encodes the primary SA receptor in plants (Wu et al., 2012), significantly decrease the severity of HLB infections. Some transgenic plants have remained disease-free for more than three years (Dutt et al., 2015). Overexpression of *NPR1* activates SA-induced defense genes in Citrus, while the interaction of NPR1 protein with *Cs*NPR3 and *Cs*TGA5 in the nucleus demonstrates that the SA network orchestrating the defense mechanism against pathogens is evolutionary conserved in Citrus (Qiu et al., 2020). Since NPR1 can induce SAR, increasing pathogen resistance in the whole plant, the expression of *NPR1* in transgenic rootstocks might be sufficient to combat *CL* in non-transgenic scions. However, a transport system may be required to deliver NPR1 to the HLB infection sites in the scions to unleash the full potential of NPR1 as a SA receptor and key regulator of SA-triggered defense.

Maintaining SA homeostasis plays a critical role in normal growth, development, and various physiological processes in plants (Rivas-San Vicente and Plasencia, 2011; Vlot et al., 2021). SA carboxyl methyltransferases (SAMTs) convert SA into methyl salicylate (MeSA) that serves as an important long-distance signal in SAR response (Chen et al., 2003; Park et al., 2007). Overexpression of *Cs*SAMT1 leads to increased concentrations of both SA and MeSA, upregulating the transcription of disease resistance genes during stress conditions. This results in reduced HLB symptoms, smaller *CL* populations, and less hyperplasia of phloem cells (Zou et al., 2021). Since MeSA is a mobile SAR signal, overexpression of SAMTs in transgenic rootstocks might be sufficient to trigger similar SAR responses throughout the entire Citrus tree, including the non-transgenic scion. These findings highlight the potential of transgenic approaches to combat HLB through the constitutive expression of SA- and SAR-related genes, which are also good candidates for rootstock-specific expression in future approaches.

## 5 Root-to-shoot transport of RNAs from transgenic donor rootstocks can fight HLB in non-transgenic scions

Plants use long-distance signaling to coordinate responses to external biotic and abiotic stress (Takahashi and Shinozaki, 2019). This long-distance communication may include metabolites, hormones, proteins, peptides, electrical signals, and small RNAs, while messenger RNAs (mRNAs) might be the most common signal vehicle (Heeney and Frank, 2023). tRNA-like structures (TLSs) are sufficient to mediate mRNA transport and are essential for the mobility of a large number of transcripts that can move through the graft junction between rootstock and scion. Transgenic mRNAs, which harbor a characteristic TLS, can move from roots into wild-type scions, where these mRNAs can be translated into proteins (Zhang et al., 2016). Hence, engineering *TLS-*mRNA hybrids in Citrus can ensure the transport of rootstock-generated transgenic transcripts to the scion, and ultimately, the translation to proteins such as NPR1 or SAMT1, while the scion remains transgene-free (**Figure 1B**).

The concept of ‘using transgenic rootstocks to produce products of non-transgenic scions in fruit trees’ was established in cherry using a RNA interference (RNAi) vector: The rootstock-to-scion transfer of the hairpin RNA (hpRNA)-derived small interfering RNAs (siRNAs) was sufficient to enhance virus resistance in the non-transgenic scion (Zhao and Song, 2014). In Citrus, expression of hpRNAs in the interstock strengthens the tolerance to Citrus psorosis virus in the whole tree (Francesco et al., 2020). This finding demonstrates that expression of RNAi vectors in Citrus rootstock or interstocks is sufficient to knock-down target genes in non-transgenic scions. In contrast to NPR1, NPR3 and NPR4 function as transcriptional co-repressors of SA-induced genes (Ding et al., 2018). Targeting *NPR3/4* by rootstock-derived RNAi could boost the SA-dependent defense response against HLB in the non-transgenic scion.

## 6 Heritable transgene-free genome editing by grafting can generate HBL-resistant Citrus

Genome editing via CRISPR/Cas9 is a powerful biotechnology, widely applied to improve traits in crop plants, including fruit trees. Currently, the prevalent method for delivering CRISPR/Cas9 components involves integration of the bacterial T−DNA into the host plant genome (Dalla Costa et al., 2020). In Citrus, CRISPR/Cas9 has been used to modify development and disease resistance genes. For instance, it was employed to reduce the expression of the *LOB1* by removing promoter enhancer regions in sweet orange and grapefruit, decreasing their susceptibility to Citrus canker disease (Peng et al., 2017; Jia et al., 2022). Similarly, the knock-out of *CsWRKY22* enhances resistance to Citrus canker (Wang et al., 2019). Although these examples demonstrate the potential of CRISPR/Cas9 genome editing in Citrus, they all employed *Agrobacterium*-mediated transformation. To obtain transgene-free Citrus mutants, removing the CRISPR/Cas9 transgene cassettes by outcrossing would be extremely time-consuming.

A more direct approach for producing transgene-free genome-edited plants is to deliver CRISPR/Cas9 components in the form of mRNA (Hu and Gao, 2023). The CRISPR/Cas9 system basically comprises two components: (i) the CRISPR-associated protein 9 (Cas9) nuclease that can generate DNA double-strand breaks at specific genomic sequences by employing (ii) guide RNAs (gRNAs), resulting in edited DNA sequences (Mali et al., 2013). Combining *Cas9* mRNA and gRNAs with TLS motifs provides the capability for RNA movement from transgenic rootstocks to distal parts in wild-type scions through graft junctions (**Figure 1C**). Here, *Cas9* mRNA is translated, and Cas9 protein with the gRNAs can produce heritable mutations at target loci (Zaman et al., 2023). This concept has recently been proved by grafting wild-type shoots of *Arabidopsis thaliana* and *Brassica rapa* onto transgenic rootstocks of *Arabidopsis thaliana* containing the TLS-modified CRISPR/Cas9 transgene cassettes. Ultimately, the flowers successfully produced transgene-free seeds with heritable mutations (Yang et al., 2023). For the combat of HLB in Citrus, we propose transgene-free CRISPR/Cas9 approaches targeting negative regulators of SA signaling and SAR response, such as *NPR3* and *NPR4*. Using a new transformation technique, which directly transforms regenerating roots (Ma H. et al., 2022), can significantly accelerate the generation of CRISPR/Cas9 donor rootstocks. After successful genome editing, transgene-free mutant scions can be grafted to transgene-free rootstocks.

## 7 Conclusion and outlook

The choice of rootstocks can provide limited resilience to HLB in Citrus scions. It remains unclear whether this partial HLB tolerance results only from significantly healthier root system indirectly promoting an overall better tree health or is provided by rootstock-originated RNAs, proteins, and/or secondary metabolites such as the stress hormone SA (Melnyk and Meyerowitz, 2015; Bowman and Joubert, 2020). Recent studies demonstrate that transgenic approaches could achieve full HLB resistance in commercial Citrus trees. Still, public and regulatory concerns about GM plants prevent their widespread commercial deployment. Using transgenic rootstocks to produce products from non-transgenic scions in Citrus and other fruit trees seems to be a reasonable alternative. Recent emerging technologies like TLS-RNA fusions could facilitate approaches with transgenic rootstocks (Zhang et al., 2016). The TLS technology can either be used to deliver the product from the transgenic rootstock to the non-transgenic scion tissue or combined with CRISPR/Cas9 genome editing to generate transgene-free mutants (Yang et al., 2023). Both strategies can be powerful tools to fight against HLB in Citrus and can even be combined, while the Citrus fruits and other products of the scion shoot will remain transgene-free. However, the TLS technology was never used in Citrus and implementation might be challenging. Several obstacles have to be overcome before TLS-based approaches can be considered for combating HLB in commercial Citrus production. First, it would be required to test, whether TLS sequences of Citrus or other plant species can promote RNA movement upwards through the graft junction. If successful, the transportation of TLS-RNAs and the translation in the scion phloem may face limitation in efficiency in comparison to the direct overexpression of the transgene in the scion. Furthermore, the choice of the transgene will influence the outcome of the TLS-based approaches. Based on recent studies, SA- and SAR-related genes seem to be good candidates to stop HLB infections at the beginning. Nevertheless, there is the risk that SA- and SAR-related approaches could aggravate the HLB symptoms, since HLB seems mostly a pathogen-triggered immune disease (Ma W. et al., 2022). Therefore, SA/SAR-independent approaches could be an alternative such as targeting the Psyllidae by TLS-delivered RNAi approaches (Shaffer, 2020; Wang, 2019). Despite of all these obstacles, we believe that the TLS technology have the potential to be a powerful tool for combating HLB and solving other Citrus threads, and this perspective should motivate us to work in this field.

## Author contributions

RG: Visualization, Writing – original draft, Writing – review & editing. XL: Writing – review & editing. SD: Writing – review & editing. QX: Writing – review & editing. RM: Conceptualization, Visualization, Writing – original draft, Writing – review & editing.
